# Valuing nurse and midwifery unit managers’ voices: a qualitative approach

**DOI:** 10.1186/s12912-021-00680-6

**Published:** 2021-09-06

**Authors:** Cate Nagle, Olumuyiwa Omonaiye, Paul N Bennett

**Affiliations:** 1 Centre for Nursing and Midwifery Research, James Cook University, 1 James Cook Drive, Queensland 4814 Townsville, Australia; 2 Townsville Hospital and Health Service, Townsville Institute of Health Research and Innovation, Townsville, Queensland Australia; 3 University of South Australia, Adelaide, South Australia Australia

**Keywords:** Nurse unit manager, Midwifery unit manager, Leadership, Heath care management, Patient safety

## Abstract

**Background:**

Nurse and Midwifery Unit Managers (NMUMs) play pivotal roles in quality patient care, nurse and midwife satisfaction and retention. NMUMs are expected to be both leaders and managers simultaneously, which may create role tension. This study aimed to explore the understanding and experience of NMUMs regarding their role; to explore what barriers and facilitators NMUMs identified to achieving the goals of their clinical area; and to explore NMUMs’ career plans.

**Methods:**

Set in Victoria, Australia, this study was guided by naturalistic inquiry using a qualitative descriptive approach. Thematic analysis was used to inductively develop core themes, which facilitated the motivations, experience and meanings underlying the data to be elaborated.

**Results:**

In all, 39 interviews were conducted with NMUMs across four hospitals. Two overarching themes were identified from the data; *system challenges* and *influences on people* and each theme had three sub-themes. In relation to system challenges, participants spoke about the structural challenges that they encountered such as financial stressors and physical infrastructure that made their work difficult. Participants felt they were unprepared for the NMUM role and had limited support in the preparation for the role. Participants also related their frustration of not being included in important decision-making processes within the hospital. Regarding their career plans, most did not envisage a career beyond that of a NMUM.

**Conclusions:**

This study of contemporary NMUMs uncovered a continued lack of investment in the orientation, professional development and support of this critical leadership and management role. There is an urgent need for targeted interventions to support and develop capabilities of NMUMs to meet the current and evolving demands of their role.

## Background

Nurse and Midwifery Unit Managers (NMUMs) play pivotal roles in quality patient care, nurse and midwife satisfaction and retention [1]. The NMUMs’ role includes change agent; coach; mentor; finance and human resource manager; clinical expert; educator; quality manager and patient advocate [2, 3]. Nurses who appraise their practice environments positively, are less likely to report burnout and leave their position, compared to nurses with a negative appraisal of their practice environments [4, 5]. Furthermore, higher level nurse-reported quality of care, has been associated with work environments where nurses have reported a feeling of being empowered to carry out their work [6].

Irrespective of the position title or experience that nurses and midwives may possess, leadership is a role that all are expected to fulfil [7, 8]. A leader inspires and influences others to act while at the same time directing the way that others act. Thus, leadership requires qualities that extend beyond management skills [9]. Conversely, a manager seeks to meet goals while following organisation rules [9].

NMUMs are expected to carry the responsibilities and exhibit the functions of a leader and a manager simultaneously [10, 11]. This involves NMUMs developing a vision and operationalising nurses and midwives towards this vision. However, being both a leader and a manager can create tensions in the NMUMs’ role [11]. Leadership has been viewed as one of the many functions of a manager, despite the fact that others might view management as a role of leadership [11]. Nevertheless, there is the contention that both management and leadership may not come together in one individual. This is because of the diverse factors in a workplace environment that make it challenging for one individual to be true to both, and that attempting to do so can result in internal conflict [12]. Hence, because of the diverse motivators and objectives in a dynamic workplace environment, there is a need for leadership training and organisational support to enhance NMUMs’ performance.

In 2016, Western Health, Victoria, Australia, initiated a review of the Unit Manager role including interviews with NMUMs to inform an organisation wide professional development of this staff group. Western Health includes several hospitals and a wide range of community based services. These services are provided mainly for the western region of Melbourne, which has a catchment population of about 800,000 people [13]. One of several information sources to inform this initiative was the NMUMs themselves. It was considered vital to the success of the overall program that NMUMs were able to describe their experiences of management and leadership within their roles and relate areas of strength and areas requiring development, for the program to be relevant and responsive. The objectives of this study were: to explore the understanding and experience of NMUMs regarding their role; to explore what barriers and facilitators NMUMs identified to achieving the goals of their clinical area; and to explore NMUMs’ career plans.

## Methods

### Research design

The study was guided by naturalistic inquiry using a qualitative descriptive approach [14]. There are three main tenets that underpin naturalistic inquiry: the phenomenon should be studied in context; the object of interest should be examined without reference to a priori theoretical frameworks and the researchers’ preconceived assumptions should be explicit; and the research is interpretive [14]. In this study, naturalistic inquiry provided an ideal method to explore NMUMs’ leadership perspectives. In order to ensure trustworthiness of qualitative data the four criteria proposed by Guba (1981) were applied in this study [15]. These four criteria are credibility, transferability, dependability and confirmability.

### Setting and participants

This study was conducted in four hospitals: Sunshine Hospital; Footscray Hospital; Sunbury Day Hospital; and Williamstown Hospital, all under the management of Western Health, Victoria, Australia. Selection criteria included all NMUMs employed during the data collection period.

Sunshine Hospital is an acute and subacute teaching hospital with approximately 600 beds. The hospital provides: elective and emergency services with a range of inpatient and outpatient services including intensive care and coronary care; acute medical and surgical services; sub-specialty medicine and surgical services; rehabilitation; aged care and palliative care services; and women’s and children’s services. Footscray Hospital is an acute and subacute teaching hospital with approximately 300 beds and provides elective and emergency services, with a range of inpatient and outpatient services including: acute general medical and surgical; intensive and coronary care; sub-specialty medicine; surgical services; rehabilitation and aged care. Williamstown Hospital is a 90-bed facility providing emergency services, surgical services, rehabilitation and geriatric evaluation and management services, renal dialysis services and community rehabilitation and transition care services. At the Sunbury Day Hospital the services provided include day medical, day surgical, day chemotherapy and haemodialysis treatment, and several specialist clinics.

### Sampling approach and recruitment of participants

A population based sampling [16] approach was used and all NMUMs were invited to participate in a face-to-face, semi-structured interview. This sampling approach was selected to ensure that all NMUMs had the opportunity to inform the professional development intervention and engaged early in the process. A distribution list of the names and contact details of NMUMs was provided to staff independent of the research team. NMUMs were sent an email describing the study with an invitation to participate in an interview from a person independent of line management. The initial email contact was followed by a phone call from a research team member to provide clarification and respond to any questions. An interview was scheduled at a mutually convenient time for those interested in participating. Participants received a hard copy of the plain language statement and consent form before providing written consent.

### Data collection

**Fig. 1:**
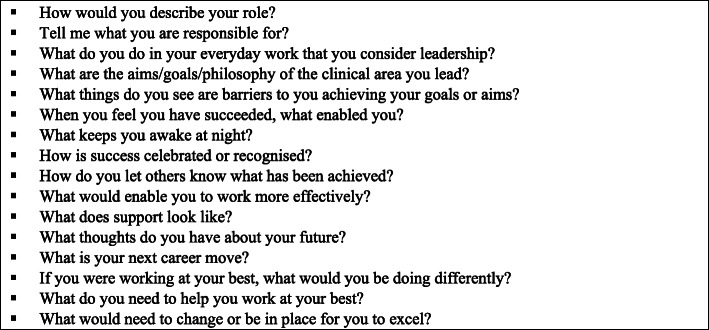
Semi structured interview Guide

Interviews were scheduled at a site convenient to the NMUM and in a private, neutral environment. A semi-structured interview schedule was used to guide discussion. During the interviews, participants were asked open-ended questions about their role and perspectives of leadership (Fig. 1). In addition, details were sought about their understanding of the barriers and facilitators to the NMUM’s role and their career plans. Data were collected using individual interviews rather than focus groups with the rationale that some individual participants may be more comfortable to share their insights in a one to one discussion. All interviews were digitally recorded with consent of the NMUMs and professionally transcribed. Transcripts were coded numerically 001–039.

### Data analysis

An inductive inference method underpinned the methodological approach that guided this study. This approach allowed new knowledge to be generated based on data that was collected [17]. Data collected from the transcripts was systematically analysed using a thematic analysis framework. Thematic analysis is also used to systematically develop core themes based on an inductive approach which allows the motivations, experience and meanings underlying the data to be elaborated upon in a straightforward manner [18]. Thematic analysis of the transcribed data was conducted using Braun and Clarke’s (2006) Framework. Initially, familiarisation of the data occurred by reading through the verbatim transcribed files. Initial themes were identified, and synthesis of collated themes was undertaken independently by members of the research team (CN & OO). Identified themes were reviewed and refined by the research team.

### Ethical considerations

 Organisational ethics approval for this study was granted by Western Health Low Risk Ethics Panel (LNR/15/WH/123). The study was conducted in accordance with the principles of the Helsinki Declaration. Written informed consent was obtained from all participants before the start of each interview. No identifying details were collected.

## Results

A total of 39 of the health services’ 51 NMUMs participated in interviews conducted between November 2015 and April 2016; NMUMs from all four sites of the health service participated. Many of the NMUMs had been employees at Western Health for many years and few had previously held senior roles in other health services. Almost all participants were female (*n* = 37, 94.9 %). The median years as an NMUM was 4 (IQR = 5.5). However, of note ten NMUM had been in the position for less than one year. The median duration of interviews was 48.5 min, ranging from minimum of 37 min to a maximum of 66 min.

Two overarching themes were identified from the data; system challenges and influences on people (Table 1) and each theme had three sub-themes.

**Table 1:** Major themes and sub-themes relating to the perspectives of Nurse Midwife Unit Mangers (NMUMs) role

Major themes	Sub-themes	Sub Sub-themes
**Systems Challenges**		
	Structural challenges	Institutional deficits-financial and physical infrastructureIn adequate support from other systems/ structures/departments.Challenges of working with hospital managementInadequate support from systems and department within the hospital management.Feelings of not being supported by superiors/hospital management.Not included in decision making within the organisation.Adherence to organisational policies and procedure
	Role preparation	Unprepared for the NMUM roleStruggling with self confidenceProfessional development for NMUMCareer plans
	Managerial and administrative functions of role	Administrative responsibilities taking time from clinical timeStaffing and skill mix issues and dealing with difficult staff.Delegation of duties
**Influences of people**		
	Leadership	Being accessibleLeading by example and taking responsibilityBuilding a functional teamTreating everyone with fairness, respect and equally.Succession plans/people developer
	Putting the patient first	Clinical responsibilities main feature of roleMultidisciplinary interactionMaintaining quality
	Personal sacrifices	Long hours of workDemand of work affecting family/personal lifeShouldering some of the personal burdens of subordinates

### System Challenges

The NMUMs related that their managerial position had increased in scope over time with wider responsibilities for patient care and management, leadership of staff, administration and management of resources. In a bid to operationalise these multidimensional roles and functions, NMUMs encountered diverse system challenges.

Three sub-themes emerged that demonstrated impediments that NMUMs faced in the day to day running of their units and made it difficult for them to achieve their goals as a NMUM (Table 1). These three sub-themes are: Structural challenges, role preparation, managerial and administrative functions of role.

#### Structural challenges

Participants talked about the structural challenges that they encountered in the form of institutional deficits, financial and physical infrastructure that made their work onerous. As one participant remarked:

*It makes it a very difficult task and [a] very time -consuming task when all I want is a medication fridge.* [023].

Participants commented on the indignation they felt seeing patients in undignified conditions because of infrastructural inadequacies in the hospital:

*Having patients paraded through the cafeteria area on their beds while they’re going from one ward to another or going to have a scan is the most awful thing to see.* [009]

Participants echoed the challenges of working with hospital management and the associated stress that comes with this working relationship:

*The other thing I find when I did have conversation with my manager is sometimes if I need something I have to go through 10, 15 questions - so why do I need - explain, provide rationale for that. So last time I said to him, well, if you really don’t trust my judgment, I don’t think I should be in the position* [009].

Inadequate support from other systems and departments within the hospital was also uppermost in the minds of most of the participants. As one participant remarked:

*….I struggle with my operations manager because < identifier removed > doesn’t get it.* [014]

Participants wanted to have some form of validation of the work they were doing and had hoped that their managers would be there for them to support their leadership efforts. Some participants expressed the feeling of not been supported by managers and/or hospital management.

*The other thing that I’ve found is … the Director of Nursing, I used to see them at least once a week. I’ve been here eight months; I haven’t seen the Director of Nursing on my ward as yet.* [009]

However, some participants expressed that they felt supported by their service group director. As a participant remarked:

*…my ops manager is very good in actually guiding me - this is my first year actually with … and I’ve learned so much from him.* [010]

Participants also talked about their frustration of not being included in important decision-making processes within the hospital:

*I find that frustrating because there isn’t that overall understanding of the workforce and it seems to me that the NEAT targets drive everything.* [014]

NEAT refers to National Emergency Access Targets. These are Australian federal government performance indicators for hospitals measuring the time from emergency presentation to discharge from the emergency department or to admission.

Despite the structural challenges that confronted the participants in their day to day running of their units, they were still focused on adhering to the policies and procedures of their organisations:

*Policies, adherence to policy, adherence to procedures, adherence to guidelines, adherence to the … values… you need to be able to communicate to people as to why those things are important and expect them to carry out those roles.* [012]

#### Role Preparation

 Most participants felt they were unprepared for the NMUM role and had had limited support in their preparation for the role.

*There’s no orientation to a NUM role, you just sort of - here’s roster on, here’s - you need to use recruit, you need to do this, you need to - there’s no structure in how you learn these tools that you’re supposed to use. There’s no formal education in how you use these tools.* [007]

In the few instances where there was orientation, participants were not satisfied with orientation process. As a result of lack of preparation and capacity building before taking on the role of NMUM position some participants struggled with self-confidence.

 Many of the participants identified that professional development and mentorship would assist them in their NMUM role. As this participant explained:

*I think if there’s going to be more expectations of the Nurse Unit Managers, then there needs to be some more education …Project management, change management, how to write a business case*. [007]

Participants were asked about their career plans and most did not envisage a career beyond that of Unit Manager or they were unsure.

*… it doesn’t seem like there’s a great future for me to go anywhere else or develop anymore or help develop anyone else. So it’s very much now just being here and doing what I’ve been doing…* [022].

Few participants identified an intent to undertake further study or a change of career.

*Just working in public health, maybe being an epidemiologist or working in epidemiology or doing research.* [028]

*I’ve thought about maybe doing some more study at some point. …Well probably just in the management side of - something that’s relevant to my role at the moment.* [026]

#### Managerial and administrative functions of role

Participants revealed that they were concerned that their administrative responsibilities were taking more time away from clinical time.

*… I think I would be very sad if I would have to give up my clinical time, because I really enjoy my clinical time. But then I’m finding that my admin days - two admin days is just not adequate for me to do everything that I need to do.* [009]

Participants identified staffing and skill mix issues and dealing with difficult staff as critical managerial function that they had to confront in order to run their units successfully.

*So obviously it’s incredibly difficult to recruit staff. It’s not just staff, it’s … trained staff……….The skill mix… high reliance and you know just for the senior staff on the unit, helping support new staff, making sure everyone is safe, making sure patients are safe, it’s been a huge workload for everyone.* [012]

To effectively run their units, few participants demonstrated the ability to delegate aspects of their NMUM role to their staff. One NMUM who did delegate responsibilities related:

*It is important to set up portfolios. So I have an infection control portfolio… I have the rostering portfolio. We have the ordering portfolio. Very important stuff… There’s an admission portfolio…. So those people all have responsibilities in their areas…. I find if you have those portfolios and those structures really early and you get the right people in them, your place runs really well. So I’ve been fairly successful as a manager* [013].

#### Influences on people

The second overarching theme was influences on people. Participants described their NMUM role in terms of investing and supporting their staff, leading and influencing their teams and being accountable and responsible for the care provided. In this theme there were three sub-themes: Leadership; putting patients first; and personal sacrifices.

#### Leadership

 In describing how they provided leadership in their everyday practice several key elements were described by the participants. Being an accessible leader was stated as very important.

*I’ve got an open-door policy. …there are managers in the past who have not allowed the staff to talk to them. I tell them where I’m going. I’m off-floor today, I’ll be - if you need, see me by 12. If it’s urgent email me and I’ll get back to you tonight after work.*[013].

Another attribute that the participants described as an example of their leadership was leading by example and taking responsibility to influence the behaviour of staff.

*I want to lead the team actually in a cohesive and a united team. So that’s really my aim and that’s why I still deliver quality care, standard of care and you know good teamwork and it’s easy for everyone.* [009]

For some participants, leadership was evidenced by building a functional team that can deliver effectively on team goals by the complementary use of skill sets.

*Finding out where staff want to work, what they don’t want to do. Trying to put people in and move them around to best suit the needs of the unit, the needs of the organisation and the staff’s needs.* [012]

The participants also identified their interactions with staff as a demonstration of their leadership. Treating others with respect and in an equitable and transparent manner were examples of leadership in their NMUM roles.

*I make it very clear that whether you’re the CEO or you’re the cleaner, I speak exactly the same. I might change my vernacular, but I do not treat them any differently basically. So everyone is treated exactly the same…* [013].

Participants demonstrated their leadership acumen by developing succession plans. This contrasted with the abrupt manner many participants experienced at the commencement of their role as NMUM. As this participant explained:

*Also developing my staff, so also going through succession planning. At the moment I have one of my nurses acting up as an ANUM, so coaching her, showing her how to do things or things like that.* [009]

#### Putting patients first

In describing their NMUM role, participants related a wide diversity of responsibilities. Participants described clinical responsibilities as a main feature of their role, particularly in terms of working with staff ‘on the floor’, to ensure that a high standard of care was provided. Participants demonstrated their mission of putting the patient first regardless of the constraining situation that they might be facing.

*Again, I need to provide care to the patients, so I’m not going to compromise patients’ care just because I have to explain at the end of the month - why did I spend more?* [009]

*So I say to my staff that they’re - they come second to the patient. At the end of the day the patient is number one. Without them I don’t have a job. So they’re a very close second, but they’re second*. [013]

For some participants multidisciplinary interaction based on communication and co-operation with other team members was crucial to ensure that patients’ interests were upheld.

*I guess in my current role a lot of my responsibility is around, I guess, communicating and negotiating with other groups of people to make sure that the patient has a good journey through the system, really.* [006]

Participants also saw patient safety as their number one priority. As this participant remarked:

*My role is first and foremost to protect the patients’ safety and to - my other part of my role is to provide leadership and direction to the unit…* [014].

#### Personal sacrifices

Some participants described the NMUM role in terms of the personal sacrifices that they had to undertake to ensure the optimal functioning of their unit. For example, participants worked long hours as a routine to ensure positive outcomes for their units. As this participant explained:

*I work longer hours, it’s for me actually to finish my own work but because I want myself to be visible on the floor to make sure that they’re all supported. I want myself to be involved in whatever they do because I always wanted them to come to me and feel free so that I know where they’re at the level of understanding, the level of - their coping mechanism.*[010].

Participants talked about how their family life was being affected because of the extra-commitment that they are putting into their job.

*I guess I’ll be honest, recently this year my partner said to me that I need to really take a long hard look at what I’m doing because I’m not home before 7pm. I’ve got a three-year-old who is starting to ignore me because I’m not there. I’m gone sometimes before he wakes up.* [012]

Despite the huge burden of responsibilities on the shoulders of the NMUMs, they were able to listen to and accommodate problems that their staff were facing in their personal lives. As this participant stated:

*…when they’ve got depression or they’re having family issues or divorces. They need to find someone - they’re very confident that I’m not going to tell anyone about their problems. I treat them exactly the same*. [008]

## Discussion

This study aimed to understand: the experience of the NMUMs’ role; the barriers to, and facilitators of, achieving their unit’s goals; and the NMUMs’ career perspectives. The findings from this study showed that many NMUMs were nurses and midwives who were appointed to their positions without an orientation or the provision of a mentor. In addition to a lack of preparation for the role, some NMUMs experienced a lack of role clarity that led to low self-confidence and feeling ill-equipped to carry out the requirements of the position. Low self-confidence on the part of the NMUMs may have cascading effects on the system [19] which may lead to reduced patient satisfaction and staff productivity [1, 19] as well as staff retention [1, 19]. Our findings suggest a need to address the NMUM preparation as a priority to contribute to staff and patient satisfaction.

Our study further revealed that targeted professional development programs on subjects such as project management, change management and business case development can enhance the capacity of NMUM to deliver effectively on their multifaceted responsibilities. Consistent with our findings, previous studies have identified confusion among NMUMs concerning the boundaries and expectations of their role, worsened by inadequate professional development opportunities relating to leadership and management in Australia [6, 20, 21], New Zealand [22], Ireland [23] and South Africa [24].

The contemporary role of NMUMs requires managerial, leadership and clinical skills [25]. Our study revealed that NMUMs were worried that their administrative responsibilities were taking more time away from their clinical leadership responsibilities. This reported drift of NMUMs from one of their core responsibilities because of increased administrative workload has the potential to compromise patient safety unless there is a restructure of roles and responsibilities. In line with our finding, evidence shows that about 65 % of a NMUM’s role is largely related to general management activities such as staff management and budgeting, with only 16 % of tasks being clinically related to patient care [25]. Therefore, it is not surprising that because of this shift in responsibilities, some have advocated that the position of the NMUM may be occupied by someone who is not a nurse [26]. This view has been espoused based on public management literature [27, 28] which alludes to the idea that any manager can manage any business [26, 27]. However, this view overlooks the everyday clinical realities that NMUMs face daily and the associated complex decisions they make relating to patient and staffing issues such as staff turnover, high part-time employment, skill mix of staff, high bed occupancy and unplanned admissions which can impact on patients’ morbidity and mortality [29]. However, if the clinical time of NMUM continues to decrease as a result of increased administrative responsibilities, the voices advocating for non-nurses to occupy the position of unit managers may become louder and even become legitimate. A recent qualitative evaluation of an intervention to reduce the administrative burden of NMUMs in Australia via the introduction of a clerical Nurse Unit Manager Support Officer position, showed that administrative support for NMUMs improved the capacities of NMUMs to undertake clinical leadership and to be strategic leaders [30].

Of note in this study was the self-sacrifices of the NMUMs. NMUMs exemplified their personal sacrifices through long hours of work and shouldering some of the personal burdens of nurses and midwives. Our study revealed that these extra commitments to their positions by NMUMs was also affecting their family life in a negative way. This culture of self-sacrifice among NMUMs is historically embedded within the nursing profession and may lead to burnout, job dissatisfaction and presenteeism [31]. A self-sacrificing image of NMUMs may also discourage prospective recruits from taking up the role of NMUMs. Thus, the detrimental over-exertion and the lack of self-care within the ranks of NMUMs needs to be addressed to reduce retention challenges. Previous studies have linked personal factors such as burnout with intent to leave [32, 33]. Both theoretical and empirical works support the use of nurses’ intention to leave as a proxy construct for actual turnover [34–36].

## Strengths and limitations

A strength of the study is the richness of the data available for analysis and the rigour of the data collection and analysis processes. Credibility, confirmability, dependability, and transferability ensure the rigour of a study [37] and in this study credibility was demonstrated through reflexivity, maintaining an audit trail, and the use of detailed descriptions in interpreting the data. Methods used in this study to establish confirmability included reflexive journaling, and a clearly identified audit trail. The transparent audit trail detailed the rationale for decisions made throughout the research process to assist establish dependability. Transferability was promoted by the large sample size and the description of the context of the study. A limitation of this study is that while it involved NMUM from four hospitals, all were from one health service which may limit transferability of findings.

## Conclusions

This study of contemporary NMUMs reveals that there continues to be a lack of investment in the orientation, professional development and support of this critical leadership and management role. Effective leadership is critical to the performance of a well-functioning hospital unit and to patient safety. Many NMUMs report feeling ill-equipped, unsure of the expectations of their role, and often overwhelmed with the demands of the position with minimal or no support from systems within the hospital. With an increasing trend to administrative and budgetary requirements within the NMUM role, the way forward is targeted interventions to support and develop capabilities of NMUMs or to introduce another model of management.

## Data Availability

Data available by contacting the corresponding author.
